# Canalicular adenoma of the tongue: report of a unique case

**DOI:** 10.11604/pamj.2021.38.337.28985

**Published:** 2021-04-07

**Authors:** Neetu Yadav, Manisha Khorate, Nigel Figueiredo

**Affiliations:** 1 Goa Dental College and Hospital, Goa, India

**Keywords:** Canalicular adenoma, salivary gland neoplasm, tongue, case report

## Abstract

Canalicular adenoma (CA) is a rare, unique benign salivary gland neoplasm, which usually involves the minor salivary glands of the upper lip, buccal mucosa and palate. It is usually seen in middle-aged or older individuals, has a female predilection, and commonly presents as a painless, slow-growing, non-ulcerated nodule or swelling. Its treatment involves surgical excision or enucleation. This case report describes a case of CA of the ventrum of the tongue diagnosed in a 41-year-old male patient, which could possibly be the first reported case of this lesion involving the tongue.

## Introduction

The term 'Canalicular Adenoma' (CA) was first used by Bauer WH and Bauer JD in 1953 [1]. Canalicular adenoma (CA) was earlier classified as a monomorphic adenoma along with other rare benign salivary gland neoplasms including basal cell adenoma, oncocytoma and warthin´s neoplasm. The term monomorphic adenoma was used to distinguish them from pleomorphic adenoma. Initially, it was believed that CA originated from terminal duct cells. The controversy about the cell of origin has led to many changes in the nomenclature and classification of CA over the years. However, in the last two editions of the World Health Organization (WHO) classification, it has been separated from other monomorphic adenomas and is considered a unique salivary gland neoplasm [1]. It represents 1-3% of all salivary gland neoplasms and occurs almost exclusively in the minor salivary glands. Clinically CA usually presents as a well-defined, painless, slow growing, mobile nodule, either firm or fluctuant on palpation. In the WHO histological classification of head and neck neoplasms, “the CA is defined as A benign salivary gland neoplasm composed of monomorphous epithelial ductal cells arranged in anastomosing cords within cell-poor vascular stroma” [2]. Based on immunohistochemistry and ultrastructural findings the cells of origin of CA are the intercalated duct luminal cells [3,4]. This paper presents a unique case of CA in a male patient which occurred on the ventrum of the tongue.

## Patient and observation

**Figure 1:**
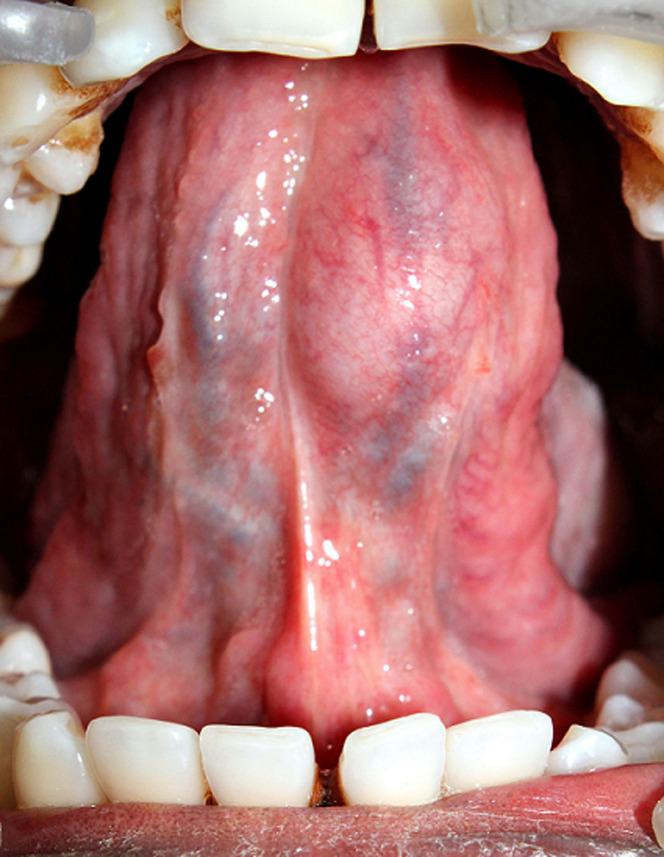
intra-oral view showing a swelling on the left ventral surface of tongue

A 41-year-old male patient reported to the out-patient department of our institution in 2019 with a complaint of swelling on the ventral surface of tongue since the last one month. The patient gave a history of a motor-vehicular accident with trauma to tongue around 45 days back. Eight (8) days later patient noticed a swelling on the ventrum of the tongue. The swelling was initially soft in consistency and became firm gradually. There was mild discomfort however, there was no difficulty in speaking or swallowing. The medical history, family history and social history were non-contributory. Intra-oral examination revealed a single, well-circumscribed, oval-shaped nodular swelling measuring approximately 1.0 x 0.8 x 0.7cm on the ventral surface of tongue on the left side of frenulum linguae. Overlying mucosa appeared normal, mildly translucent, with no visible pulsations. There was mild deviation of frenulum linguae to the right side (Figure 1). On palpation, the swelling was non-tender, firm, with a smooth surface and well defined margins. It was freely mobile, non-compressible, non-fluctuant and non-pulsatile. There was no restriction of tongue mobility. No cervical lymphadenopathy was evident. Based on the clinical appearance, a provisional diagnosis of extravasation mucocele was given, with a differential diagnosis of pleomorphic adenoma, traumatic neuroma, schwannoma and myofibroma.

**Figure 2:**
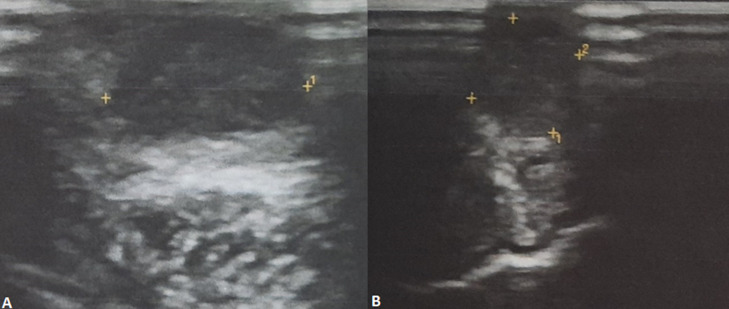
(A,B) ultrasonography examination showing a hypoechoic well-defined lesion with smooth margins within substance of the tongue

**Figure 3:**
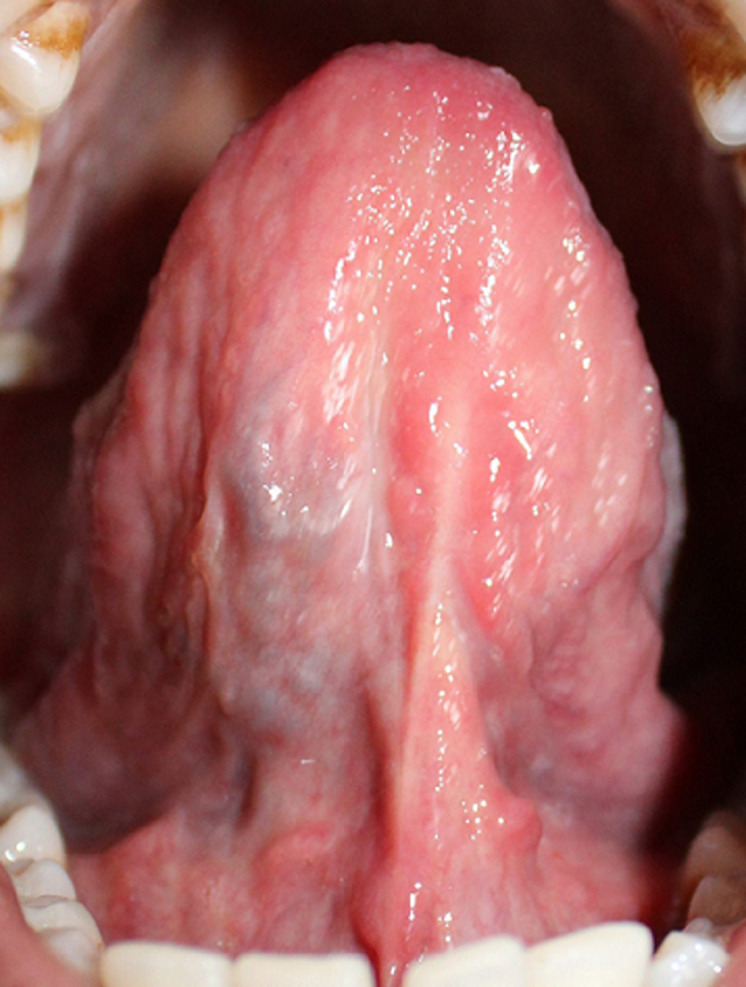
postoperative intra-oral view showing complete healing

**Figure 4:**
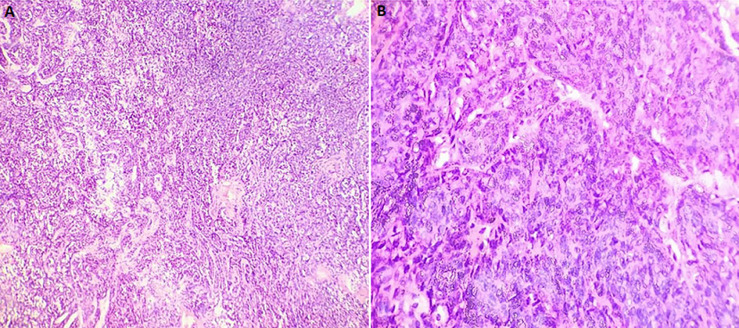
histological appearance of tumour showing eosinophilic columnar cells with regular nuclei forming canal like ductal structures [haematoxylin and eosin stained sections (A) 10x, (B) 40x]

Ultrasonography (USG) examination revealed a hypoechoic, well defined, benign appearing lesion with smooth margins present within the substance of the tongue. There was no internal vascularity seen, even with low flow settings (Figure 2). Hamartoma and Solitary Neurofibroma were considered in the differential diagnosis after USG. Based on the clinical features and USG report, the patient was advised surgical excision of the lesion. The lesion was excised under local anesthesia. The excised specimen showed a brownish white colour and was firm in consistency. Postoperative healing was uneventful (Figure 3). Histopathological examination revealed a well circumscribed benign epithelial neoplasm comprising a single layer of columnar cells along with formation of canal like ductal structures. Individual cells were eosinophilic with regular nuclei and absence of pleomorphism (Figure 4). Based on the histopathological analysis, a final diagnosis of canalicular adenoma of minor salivary glands was given. The patient gave consent for his images and clinical information to be published in a journal. The patient was explained that his name would remain anonymous and only the intraoral photographs would be used.

## Discussion

Canalicular adenoma was historically, “considered to be part of the spectrum of morphologic changes seen in basal cell adenoma. However, it is now thought to be a separate entity due to its distinctive clinical, morphologic, immunohistochemical (IHC), and ultrastructural characteristics” [4]. The exact etiology of the lesion is still unknown [5]. It typically occurs in adults during fourth to the ninth decade of life. The mean age of occurrence is seventh decade. It usually shows a female predominance, [6] with a male to female ratio of 1: 1.8 [7,8]. The upper lip is the most common site of occurrence (68-81%), followed by the buccal mucosa (16%) and hard palate (10%) [4,9,10]. Peraza AJ, Wright J, Gómez R (2017) conducted a systematic review of 356 cases of CA (for whom data was available). “They found that the cases had an average age of 66.3 years (range from 26 to 85 years) and 64% of the patients were females. A majority of cases were found in the upper lip (55.4%), followed by the hard palate (14.5%), buccal mucosa (12%), and upper lip and buccal mucosa (11.4%). Rare cases were also observed in the parotid glands (1.1%), oesophagus (0.3%), retromolar area (0.3%), soft palate (0.3%), with no case noted on the tongue” [3]. The present case was diagnosed in a 41-year-old male, and occurred on the ventrum of tongue. The age of our patient falls within the starting spectrum of the age range previously reported for CA. However, a literature search in PubMed did not reveal any previous case of CA reported to occur on the tongue, thus making the present case possibly the first case of canalicular adenoma to be reported involving the tongue.

Clinically, CA lesions are characterized by a well circumscribed, small (0.4-2cm in largest dimension), non-ulcerated, non-tender swelling or nodule that can be firm or fluctuant [3,11]. The most common presentation is a single nodule; however, it can occur as multifocal lesion, with nodules presenting distinctly away from the main neoplasm [3,11,12]. “They typically present as a slow-growing, painless, freely moveable, compressible swelling in the mucosa and submucosal tissue” [4]. The colour of the overlying mucosa is generally normal but it may have a bluish hue and mimic a mucocele [13]. This was noted in the present case, which appeared as a nodular translucent swelling, clinically resembling a mucocele. On microscopic examination, this neoplasm is composed of a single layer of cuboidal or columnar cells which may be parallel, forming long canals, columns or cords [7]. The histopathological examination of present case also revealed columnar cells in single layer forming canal like ductal structures. Sometimes rows of cells are closely approximated and appear as a double row of cells [7]. “Periodic separation of the bi-layered epithelium is frequently found which is commonly referred to as a “beading pattern” [6]. Occasionally, the cords may enclose cystic spaces of different sizes which may be filled with eosinophilic coagulum [7]. The cells show ovoid, hyperchromatic nuclei with pale to moderate staining of the cytoplasm [6]. The supporting stroma is loose and fibrillary with presence of delicate vascularity [7]. Local surgical excision is the definitive treatment of choice for CA. Surgical excision with a thin margin of apparently normal tissue is adequate even if a biopsy is not performed [6]. On excision, CA are usually light tan to brown and well circumscribed, which was noted in our case. Recurrence is rare and has been reported in small number of cases. Our case has been under follow-up for the last six months and no evidence of recurrence has been noted. Multifocal lesions are thought to have a higher rate of recurrence. Benign behavior is the usual presentation and there are no reports of malignant transformation [14].

## Conclusion

Canalicular adenoma most commonly occurs on the upper lip, followed by the buccal mucosa and palate with no cases reported on the tongue. However, as seen in the present case, despite its rarity, CA can also be considered in the differential diagnosis of swellings of the tongue. While surgical excision is the treatment of choice, some lesions have a tendency to be multifocal, in which case early diagnosis is vital in its management.
